# IgG4-related inflammatory pseudotumor presenting with refractory seizures: a diagnostic pitfall

**DOI:** 10.1055/s-0046-1822633

**Published:** 2026-06-25

**Authors:** Polyana Favero Ferreira Caetano, Vitor Ribeiro Paes, Carlos Eduardo Roelke, Lívia Almeida Dutra

**Affiliations:** 1 Hospital Israelita Albert Einstein, Instituto de Ensino e Pesquisa, São Paulo SP, Brazil.; 2 Hospital Israelita Albert Einstein, Departamento de Patologia, São Paulo SP, Brazil.; 3 Hospital do Servidor Público Estadual, São Paulo SP, Brazil.

**Keywords:** IgG4, Seizures, Immunohistochemistry

## Abstract

Immunoglobulin G4-related disease (IgG4-RD) is a systemic fibroinflammatory condition that can mimic malignancy, infection, or other autoimmune disorders, causing significant diagnostic challenges. Neurological involvement is uncommon, most frequently presenting as hypertrophic pachymeningitis or hypophysitis, but it may also manifest as inflammatory ophthalmic or cerebral pseudotumors. We herein describe a complex case of neurological IgG4-RD with a 10-year disease course, initially presenting with focal epilepsy and later evolving into a tumefactive brain lesion. This case highlights key clinical lessons: neurological IgG4-RD may remain subclinical, with only functional symptoms preceding structural abnormalities; the histopathological demonstration of storiform fibrosis and IgG4-positive plasma cell infiltration remain the diagnostic cornerstone; and systemic assessment is essential due to frequent multiorgan involvement. Finally, we discuss strategies to interpret brain-biopsy findings in inflammatory brain disorders and emphasize the importance of maintaining a high index of suspicion for IgG4-RD in atypical presentations.

## TEACHING POINTS

1. Patients with IgG4-related disease (IgG4-RD) may present inflammatory brain lesions and epilepsy.
2. The diagnosis is based on biopsy of the affected organs and on the identification of classic findings such as storiform fibrosis, lymphoplasmacytic infiltrates, and obliterative phlebitis.
3. A high IgG4-positive/IgG-positive plasma cell ratio (> 40%) is critical for diagnosis.

## CLINICAL VIGNETTE

A previously healthy 43-year-old woman developed focal motor seizures characterized by tonic movements of the right arm, progressing to bilateral tonic-clonic seizures. Initially, a brain magnetic resonance image (MRI) scan and an electroencephalogram (EEG) showed no abnormalities. She was started on valproate, with occasional focal seizures.

After 10 years, the seizure semiology extended, involving the right leg as well. She denied headaches, weight loss, or fever. A neurological examination revealed normal strength (5/5), brisk reflexes, and absent plantar responses, without sensory or cerebellar deficits. The extraocular movements were normal, and no abnormal movements were observed.

**Figure 1:**
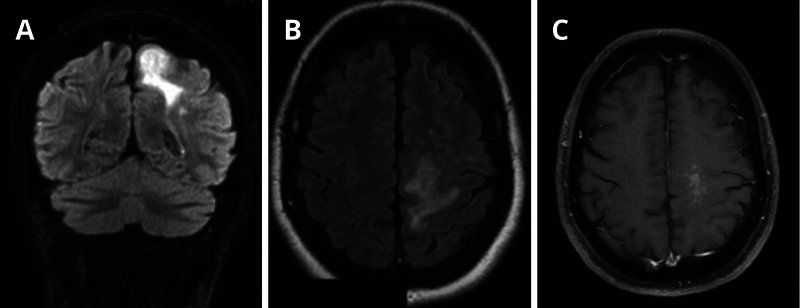
( **A** ) Coronal fluid-attenuated inversion recovery (FLAIR) sequence showing a hyperintense lesion in the left precentral gyrus with surrounding vasogenic edema. ( **B** ) Axial FLAIR sequence demonstrating a hyperintense lesion involving the cortex and subcortical white matter in the same region. ( **C** ) Postcontrast T1-weighted image revealing focal parenchymal enhancement in the left precentral gyrus, consistent with active inflammatory involvement related to immunoglobulin 4-related disease.

**Figure 2:**
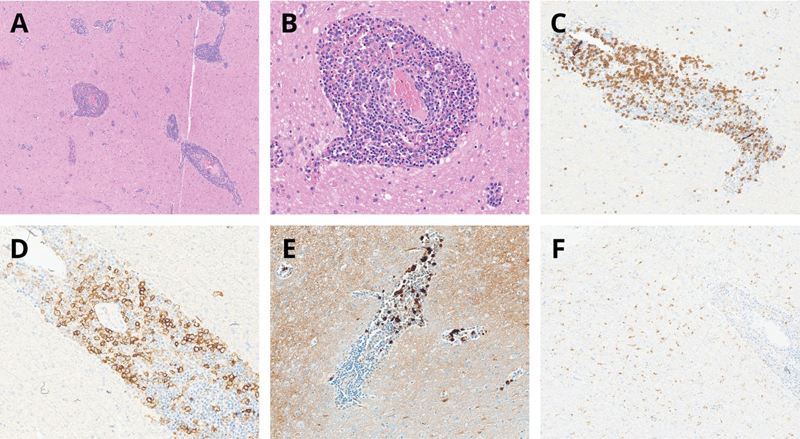
( **A,B** ) Hematoxylin and eosin (H&E) staining: cortical and white matter nervous tissue with perivascular cuffing, composed of T-cell lymphocytes CD3 ( **C** ) and plasma cells CD138 ( **D** ), most of them producing IgG4 ( **E** ). There is also microglial activation with CD68 staining ( **F** ), without nodule formation.

A new brain MRI scan demonstrated a gadolinium-enhancing lesion in the left precentral gyrus ( Figure 1 ). Suspecting a glioma, an open biopsy was performed. Histopathology excluded glial neoplasms and showed a dense fibroinflammatory infiltrate with storiform fibrosis and perivascular lymphoplasmacytic inflammation. Immunohistochemistry revealed > 30 IgG4-positive plasma cells per high-power field (hpf) and an IgG4/IgG ratio > 40%, confirming IgG4-RD ( Figure 2 ). A whole-body computed tomography (CT) scan revealed pulmonary ground-glass opacities. The patient was treated with prednisone 60 mg/day (1 mg/kg/day) and azathioprine 200 mg/day (3 mg/kg/day), along with levetiracetam, topiramate, and clobazam for seizure control.

Four months later, she developed secondary amenorrhea, though a pituitary MRI scan was normal. One year later, she presented involuntary arrhythmic movements of the right foot, consistent with paroxysmal dyskinesia, accompanied by mild weakness and hyperreflexia. A new MRI scan showed enlargement of the previous lesion. Video-EEG was normal, and electroneuromyography (EMG) showed normal motor-unit morphology with reduced recruitment and no evidence of active or chronic denervation, consistent with an upper motor neuron lesion. She received intravenous (IV) methylprednisolone (1,000 mg for 5 days) followed by rituximab 2,000 mg, achieving disease control and complete improvement of the brain lesion on MRI.

## FROM PRESENTATION TO RESOLUTION: LESSONS LEARNED

### What is IgG4-related disease?

Immunoglobulin G4-related disease is a systemic, immune-mediated fibroinflammatory disorder characterized by tumefactive lesions, lymphoplasmacytic infiltrates rich in IgG4-positive plasma cells, and variable fibrosis. It is more prevalent in men older than 50 years of age. 1 The clinical manifestations are diverse, depending on the affected organs, and they can include headache, abdominal or lumbar pain, jaundice, focal neurological deficits, visual changes, and constitutional symptoms such as weight loss, arthralgia, and fatigue. 1 Allergic diseases and tumefactive lesions are two common findings in any type of IgG4-RD. 2

The disease follows an indolent course, with relapses and remissions. The early stages may be characterized by microscopic inflammatory changes insufficient to produce detectable abnormalities on conventional MRI. Nevertheless, it often mimics malignancy, infection, or other autoimmune diseases, leading to diagnostic delays. 1 2 Clinical severity varies, ranging from mild single-organ disease to multisystem involvement with substantial morbidity. 1 The common systemic manifestations of IgG4-RD include: 3 4 5

- Pancreatic involvement (autoimmune pancreatitis, AIP): the most frequent manifestation, affecting 40% of the patients with systemic IgG4-RD, who may present abdominal pain (with a pancreatic mass), jaundice, and elevated liver enzymes;
- Biliary involvement (sclerosing cholangitis): approximately 20% of the patients with IgG4-RD present involvement of the bile ducts, leading to a form of sclerosing cholangitis; and
- Salivary-gland involvement (sialadenitis): it occurs in about 40% of the patients, and the symptoms include pain, swelling, xerostomia, and symmetrical enlargement.

The disease may involve the lungs and pituitary gland. Pulmonary involvement ranges from asymptomatic radiological findings to cough, dyspnea, hemoptysis, and respiratory discomfort. 5 In IgG4-related hypophysitis, the most frequent presentation is panhypopituitarism, which can manifest as malaise, loss of appetite, weight loss, polyuria, polydipsia, amenorrhea, and reduced libido. 6 7

Laboratory tests should be ordered, either to complement the diagnostic workup or to monitor the patient throughout treatment. These include total IgG and IgG subclass measurements (particularly serum IgG4), IgE, complement components (C3, C4, and CH50), and serum protein electrophoresis (SPEP). 1 6 7 Additional tests, such as complete blood count, renal and hepatic function panels, amylase and lipase levels, as well as others, depending on the organs involved (such as thyroid function tests, hemoglobin A1c, and pituitary hormones), are also be indicated. 1

Common alterations may include hypocomplementemia (decreased C3, C4, or CH50), elevated serum levels of IgE and multiple IgG subclasses (including IgG4, IgG1, and IgG2), as well as increased inflammatory markers such as C-reactive protein (CRP) and erythrocyte sedimentation rate (ESR). 1 2 6 7 8 Importantly, elevated serum IgG4 levels are not disease-specific—they may also occur in other autoimmune diseases, such as rheumatoid arthritis, Castleman's disease, and antineutrophil cytoplasmic antibody (ANCA)-associated vasculitis, as well as in conditions such as bronchiectasis, parasitic infections, primary sclerosing cholangitis, and malignancies (including pancreatic tumors). 7 9 Therefore, while serum IgG4 levels are important, they should not be interpreted alone. 10

In addition to the laboratory evaluation, CT, ultrasonography, and MRI help ascertain organ involvement. 1 2 6 7 Positron-emission tomography-CT (PET-CT) may detect metabolically-active inflammatory lesions in extraneural organs that are clinically silent or poorly-characterized on conventional imaging, thereby guiding the selection of safer and more accessible biopsy sites. In addition, PET-CT can be useful in the assessment of the extent of the systemic disease and in monitoring the response to immunosuppressive therapy. 1 4 6 7 Finally, histopathological studies are essential in the diagnostic workup, as tissue biopsy demonstrating infiltration of IgG4-positive plasma cells represents a diagnostic hallmark. 1 2 6 7 11

The two major criteria for the disease, the American College of Rheumatology/European League Against Rheumatism (ACR/EULAR) 2019 criteria 2 and the 2020 Japanese criteria, 12 incorporate serum IgG4 quantification, as well as radiological and histopathological findings. In the ACR/EULAR classification, the entry criterion must first be met—the involvement of at least one organ commonly affected by IgG4-RD. Subsequently, a set of exclusion criteria is assessed to rule out alternative diagnoses. If no exclusion criteria are met, the final step involves scoring a combination of histopathological, immunohistochemical, serological, and radiological findings; each assigned a specific weight. A total score ≥ 20 points confirms the classification as IgG4-RD 2 ( Table 1 ).

**Table 1:** Categorical assessment or numeric weight 2

**Step 1. Entry criteria**	
Characteristic* clinical or radiological involvement of a typical organ (eg., pancreas, salivary glands, bile ducts, orbits, kidney, lung, aorta, retroperitoneum, pachymeninges, or thyroid gland [Riedel's thyroiditis]), or pathological evidence of an inflammatory process accompanied by a lymphoplasmacytic infiltrate of uncertain etiology in one of these same organs	**Yes or no**
**Step 2. Exclusion criteria: domains and items**	
**Clinical**	**No**
• Fever	
• No response to glucocorticoids	
**Serological**	
• Leukopenia and thrombocytopenia with no explanation	
• Peripheral eosinophilia	
• Positive antineutrophil cytoplasmic antibody (specifically against proteinase 3 or myeloperoxidase)	
• Positive SSA/Ro or SSB/La antibody	
• Positive double-stranded DNA, RNP, or Sm antibody	
• Other disease-specific autoantibodies	
• Cryoglobulinemia	
**Radiological**	
• Known radiological findings with suspicion of malignancy or infection	
• Rapid radiological progression	
• Long-bone abnormalities consistent with Erdheim-Chester disease	
• Splenomegaly	
**Pathological**	
• Cellular infiltrates suggesting malignancy that have not been sufficiently evaluated	
• Markers consistent with inflammatory myofibroblastic tumor	
• Prominent neutrophilic inflammation	
• Necrotizing vasculitis	
• Prominent necrosis	
• Primarily-granulomatous inflammation	
• Pathological features of macrophage/histiocytic disorder	
**Known diagnosis of the following:**	
• Multicentric Castleman disease	
• Crohn's disease or ulcerative colitis (if only pancreatobiliary disease is present)	
• Hashimoto's thyroiditis (if only the thyroid is affected)	
⮚ If the case meets the entry criteria and does not meet any exclusion criteria, proceed to step 3	
**Step 3. Inclusion criteria: domains and items§**	
**Histopathology**	
Uninformative biopsy	0
Dense lymphocytic infiltrate	4
Dense lymphocytic infiltrate and obliterative phlebitis	6
Dense lymphocytic infiltrate and storiform fibrosis with or without obliterative phlebitis	13
**Immunostaining¥ (0–16, as follows:)**	
The IgG4 + :IgG+ ratio ranges from 0–40% or indeterminate, and the number of IgG4+ cells/hpf is 0 to 9‡	0
1) The IgG4 + :IgG+ ratio is ≥ 41% and the number of IgG4+ cells/hpf ranges from 0–9 or is indeterminate; or 2) the IgG4 + :IgG+ ratio ranges from 0–40% or is indeterminate and the number of IgG4+ cells/hpf is ≥ 10 or indeterminate	7
1) The IgG4 + :IgG+ ratio ranges from 41–70% and the number of IgG4+ cells/hpf is ≥10; or 2) the IgG4 + :IgG+ ratio is ≥ 71% and the number of IgG4+ cells/hpf ranges from 10–50	14
The IgG4 + :IgG+ ratio is ≥ 71% and the number of IgG4+ cells/hpf is ≥ 51	16
**Serum IgG4 concentration**	
Normal or unknown	0
⮚ Normal but < 2 times the upper limit of normal	4
2–5 times the upper limit of normal	6
> 5 times the upper limit of normal	11
**Bilateral lacrimal, parotid, sublingual, and submandibular glands**	
No glands involved	0
1 set of glands involved	6
≥ 2 sets of glands involved	14
**Chest**	
Not checked or none of the items listed is present	0
Peribronchovascular and septal thickening	4
Paravertebral band-like soft tissue in the thorax	10
**Pancreas and biliary tree**	
Not checked or none of the items listed is present	0
Diffuse pancreas enlargement (loss of lobulations)	8
Diffuse pancreas enlargement and capsule-like rim with decreased enhancement	11
Pancreas (either of the items above) and biliary tree involvement	19
**Kidney**	
Not checked or none of the items listed is present	0
Hypocomplementemia	6
Renal pelvis thickening/soft tissue	8
Bilateral kidney cortex low-density areas	10
**Retroperitoneum**	
Not checked or none of the items listed is present	0
Diffuse thickening of the abdominal aortic wall	4
Circumferential or anterolateral soft tissue around the infrarenal aorta or iliac arteries	8
**Step 4: Total inclusion points**	
A case meets the classification criteria for IgG4-RD if the entry criteria are met, no exclusion criteria are present, and the total is ≥ 20 points	

Abbreviations: hpf, high-power field; IgG4-RD, IgG4-related disease; RNP, ribonucleoprotein.

Notes: ^*^ Refers to enlargement or tumor-like mass in an affected organ except in 1) the bile ducts, where narrowing tends to occur, 2) the aorta, where wall thickening or aneurysmal dilatation is typical, and 3) the lungs, where thickening of the bronchovascular bundles is common.

¶ If entry criteria are not fulfilled, the patient cannot be further considered for classification as having IgG4-related disease (IgG4-RD).

◊ If exclusion criteria are met, the patient cannot be further considered for classification as having IgG4-RD.

§ Only the highest-weighted item in each domain is scored.

¥ Biopsies from lymph nodes, mucosal surfaces of the gastrointestinal tract, and skin are not acceptable for use in weighting the immunostaining domain.

‡ “Indeterminate” refers to a situation in which the pathologist is unable to clearly quantify the number of positively staining cells within an infiltrate yet can still ascertain that the number of cells is at least 10/high-power field (HPF). For a number of reasons, most often pertaining to the quality of the immunostain, pathologists are sometimes unable to count the number of IgG4+ plasma cells with precision yet even so, can be confident in grouping cases into the appropriate immunostaining result category.

The 2020 Japanese criteria 12 follow a similar approach, consisting of three domains: clinical and radiological findings and serological and histopathological diagnoses. A case is classified as: *definite* when all three domains are fulfilled; *probable* when domains 1 and 3 are present; and *possible* when domains 1 and 2 are met. Table 2 provides a complete description of this classification system. 12

**Table 2:** 2020 revised comprehensive diagnostic criteria for IgG4-RD 2

Item 1	Clinical and radiological features
	One or more organs show diffuse or localized swelling or a mass or nodule characteristic of IgG4-RD. In single-organ involvement, lymph-node swelling is omitted.
**Item 2**	**Serological diagnosis**
	Serum IgG4 levels > 135 mg/dL
**Item 3**	**Pathological diagnosis**
Positivity for two of the following three criteria:	1. Dense-lymphocyte and plasma-cell infiltration with fibrosis.
	2. Ratio of IgG4-positive plasma cells/IgG-positive cells > 40% and the number of IgG4-positive plasma cells > 10 per high-power field
	3. Typical tissue fibrosis, particularly storiform fibrosis, or obliterative phlebitis
**Diagnosis**	
Definite:	Items 1 + 2 + 3
Probable: 1) + 3):	Items 1 + 3
Possible: 1) + 2)	Items 1 + 2

Abbreviations: IgG, immunoglobulin G; IgG4, immunoglobulin G4; IgG4-RD, immunoglobulin G4-related disease.

In the case herein reported, the patient demonstrated pulmonary disease, evidenced by ground-glass infiltrates, and possible pituitary dysfunction manifested by amenorrhea with reduced gonadotropins, despite a normal MRI scan. These findings reinforce the systemic nature of IgG4-RD and the need to investigate extraneurological involvement in patients presenting neurological symptoms.

### Neurological manifestations of IgG4-RD

Immunoglobulin G4-related disease is characterized by a fibroinflammatory process driven by dysregulated immune responses involving type-2 helper T cells (Th2) and regulatory T cells, with overproduction of interleukin-4 (IL-4), (IL-10), and transforming growth factor-Beta (TGF-β). In the central nervous system (CNS), this immune activity targets meningeal, pituitary, or parenchymal tissues, resulting in thickening, mass-like lesions, or compressive symptoms. 1 6 7 The most frequent CNS manifestations are hypertrophic pachymeningitis (∼ 1.3% of the systemic cases) and hypophysitis (∼ 1.5%). 7 Less commonly, the disease presents as an inflammatory CNS pseudotumor, often mimicking neoplasia. 7 Perineural involvement has also been reported, most frequently in the orbital and paravertebral regions, causing acute and subacute neuropathies. 7

The clinical presentation of CNS involvement depends on the affected site. In a review of 33 biopsy-confirmed cases of IgG4-related hypertrophic pachymeningitis, headache was the most frequent symptom (67%), followed by cranial nerve palsies (33%), visual disturbances (21%), motor weakness (15%), limb numbness (12%), hearing loss (9%), seizures (6%), and cognitive decline (3%). 7 13 Pseudotumoral forms may involve the meninges, ventricles, parietotemporal cortex, pituitary gland, cranial nerves, or spinal cord, and symptoms typically arise from compressive effects, depending on location. 7

Neuroimaging and CSF analysis are key components of the diagnostic evaluation in neuro-IgG4-RD. The MRI scans typically show dural thickening with contrast enhancement or mass-like lesions resembling meningioma or lymphoma. 7 The cerebrospinal fluid (CSF) findings are nonspecific, and lymphocytic pleocytosis and mild elevated protein are relevant for the differential diagnosis. More recently, intrathecal IgG4 synthesis—evidenced by elevated CSF IgG4 levels or a high IgG4 index—has been proposed as a diagnostic marker. 7

Data on long-term survival in IgG4-RD with CNS involvement remains limited. A systematic review 14 of IgG4-related pachymeningitis reported mortality below 1%, with relapses occurring in approximately 40% of the patients during a median follow-up of 9 months. Overall, prognosis appears favorable in the short term, particularly with immunosuppressive therapy, although fibrotic progression may lead to irreversible neurological deficits. The limited number of long-term studies spotlight the need for further research to clarify survival and relapse predictors in neuro-IgG4-RD.

### When should IgG4-RD be suspected by neurologists according to the histopathological results and how to manage the disease?

Histopathological confirmation is fundamental, especially in cases of mimicking neoplasia or granulomatous diseases. Three key histological hallmarks define IgG4-RD in most of the organ sampled: 1 11 dense polyclonal lymphoplasmacytic infiltrate enriched in IgG4-positive plasma cells; storiform (“woven mat”) fibrosis pattern; and obliterative phlebitis, characterized by venous-channel destruction due to inflammatory infiltrates.

The diagnostic sensitivity of tissue biopsy varies according to the organ involved. The best option is to biopsy the symptomatic site; however, biopsies of lymph nodes should be interpreted with caution, because they rarely develop storiform fibrosis and IgG4-positive plasma cells may be present because of alternative diagnosis, resulting in poor specificity of this finding. The most specific histological finding seems to be obliterative phlebitis. 7 Because the gastrointestinal tract (GTI) is seldom affected in IgG4-R and contains a high concentration of IgG4+ plasma cells, GTI biopsies should not be used to diagnose IgG4-RD.

Additional stains, such as elastin, may assist in identifying obliterated vessels. However, interpretation requires awareness of tissue-specific thresholds. The absolute number of IgG4-positive plasma cells per hpf varies depending on the organ, ranging from > 10/hpf in the meninges to > 100/hpf in the skin. Regardless of tissue, a ratio of IgG4-positive/IgG-positive plasma cells exceeding 40% is considered highly suggestive of the disease. Importantly, patchy fibrosis can create diagnostic pitfalls in small biopsies, potentially leading to underrecognition of the characteristic pattern. 1 7

The therapeutic goal is to achieve immunosuppression to control inflammation and prevent fibrosis. Glucocorticoids remain the first-line treatment, with prednisone typically initiated at approximately 0.5 mg/kg/day and gradually tapered throughout several months. 1 6 7 In neurological presentations, the initial therapy involves IV methylprednisolone to minimize irreversible damage. 7 For relapsing or refractory disease, rituximab ,2000 mg has shown efficacy 10 and is now often used as a first-line agent or steroid-sparing agent. More recently, inebilizumab (300mg IV every 15 days; 2 doses) has been approved as another B-cell-depleting option, with promising results. 10 15 Other oral maintenance therapies may include azathioprine and mycophenolate mofetil.

In conclusion, the case herein reported illustrates the diagnostic and therapeutic challenges of IgG4-RD with neurological involvement. The patient initially presented with focal seizures and normal brain imaging, delaying the suspicion of an underlying inflammatory disorder. Over time, she also developed systemic features, including pulmonary disease and pituitary dysfunction, before biopsy confirmation.

Although a 10-year interval is uncommon, delayed radiological manifestation has been reported in this disease, because of its course. Thus, functional neurological symptoms, such as seizures, may precede overt structural lesions by several years.

Consequently, clinicians should maintain a high index of suspicion for IgG4-RD in patients with chronic, unexplained neurological symptoms, particularly when imaging or histology suggests inflammation or fibrosis. Recognition of characteristic histopathological features—storiform fibrosis and IgG4-rich infiltrates—is essential to avoid misdiagnosis.

Finally, the case herein reported reinforces the importance of systemic evaluation to detect multiorgan involvement, and it highlights the role of immunosuppressive therapy, including rituximab and inebilizumab, in achieving remission and preventing irreversible damage.
